# A computational model of *Pseudomonas syringae* metabolism unveils a role for branched-chain amino acids in Arabidopsis leaf colonization

**DOI:** 10.1371/journal.pcbi.1011651

**Published:** 2023-12-27

**Authors:** Philip J. Tubergen, Greg Medlock, Anni Moore, Xiaomu Zhang, Jason A. Papin, Cristian H. Danna

**Affiliations:** 1 Department of Biology, University of Virginia, Charlottesville, Virginia, United States of America; 2 Department of Biomedical Engineering, University of Virginia, Charlottesville, Virginia, United States of America; US Army Medical Research and Materiel Command: US Army Medical Research and Development Command, UNITED STATES

## Abstract

Bacterial pathogens adapt their metabolism to the plant environment to successfully colonize their hosts. In our efforts to uncover the metabolic pathways that contribute to the colonization of *Arabidopsis thaliana* leaves by *Pseudomonas syringae* pv *tomato* DC3000 (*Pst* DC3000), we created iPst19, an ensemble of 100 genome-scale network reconstructions of *Pst* DC3000 metabolism. We developed a novel approach for gene essentiality screens, leveraging the predictive power of iPst19 to identify core and ancillary condition-specific essential genes. Constraining the metabolic flux of iPst19 with *Pst* DC3000 gene expression data obtained from naïve-infected or pre-immunized-infected plants, revealed changes in bacterial metabolism imposed by plant immunity. Machine learning analysis revealed that among other amino acids, branched-chain amino acids (BCAAs) metabolism significantly contributed to the overall metabolic status of each gene-expression-contextualized iPst19 simulation. These predictions were tested and confirmed experimentally. *Pst* DC3000 growth and gene expression analysis showed that BCAAs suppress virulence gene expression *in vitro* without affecting bacterial growth. *In planta*, however, an excess of BCAAs suppress the expression of virulence genes at the early stages of infection and significantly impair the colonization of Arabidopsis leaves. Our findings suggesting that BCAAs catabolism is necessary to express virulence and colonize the host. Overall, this study provides valuable insights into how plant immunity impacts *Pst* DC3000 metabolism, and how bacterial metabolism impacts the expression of virulence.

## Author summary

Bacterial infections are a leading cause of crop yield reduction worldwide. Plants utilize an intricate immune system to prevent invading microbes from establishing an infection. Part of this plant immune response results in changes in the metabolite composition that impact the ability of microbes to produce disease. To study these complexities, we created iPst19, an ensemble of 100 metabolic models of the bacterial pathogen *Pseudomonas syringae*. iPst19 can predict conditional metabolic differences in *P*. *syringae* exposed to naïve plants, where infections progress rapidly, versus pre-immunized plants, where infections are significantly restricted by plant immunity. iPst19 growth simulations predicted branched-chain amino acid metabolism as a key factor that contributes to regulating the expression of bacterial virulence, a necessary condition to produce infections. We have corroborated predictions made by iPst19 with in vivo experiments by supplementing plants with amino acids to reduce the infectivity of *P*. *syringae*, revealing another critical aspect of pathogen-induced defense mechanisms. Importantly, we have created a new computational tool to allow a deeper understanding of the *Arabidopsis thaliana*–*P*. *syringae* interactions, the most studied plant-pathogen interaction system.

## Introduction

*Pseudomonas syringae* is a pathogenic bacterium that can infect a wide range of plant species, often in a species-specific interaction [1]. Based on its host range, more than 60 different pathovars have been identified [2]. In addition to causing disease in tomatoes, *Pseudomonas syringae* pv. *tomato* DC3000 (*Pst* DC3000) can infect the model plant *Arabidopsis thaliana* [3]. The genetic tractability of the *Pst* DC3000-*Arabidopsis* pathosystem has facilitated the discovery of molecular mechanisms underlying plant defense and bacterial disease that have been translated to distant plant species, including crops [1].

*Pst* DC3000 is an endophytic pathogen that colonizes the leaf apoplast (LA), the intercellular spaces of the leaf mesophyll. The LA is partially filled with an aqueous solution rich in proteins, sugar polymers, free hexoses, free amino acids, and other plant-synthesized metabolites that can regulate *Pst* DC3000 growth [4]. The concerted activity of membrane transporters and LA resident enzymes regulate the concentration of each metabolite in basal conditions and in response to stress [5,6].

Once inside the LA, *Pst* DC3000 subverts early inducible plant defense responses that would otherwise restrict its growth and prevent the onset of disease [7–9]. To suppress plant immunity, *Pst* DC3000 uses the Type-3 Secretion System (T3SS) to inject effector proteins into host cells. The expression of T3SS genes is modulated by the transcriptional regulator *hrpL*. In addition, the phytotoxin coronatine, whose synthesis requires the enzyme coronafacate ligase (*cfl*), is a key element in the *Pst* DC3000 virulence repertoire [7,10,11]. The modulation of the expression of virulence factors depends on nutritional and environmental cues that quickly change during leaf colonization due to the activation of plant immunity.

Plants perceive invading microbes via the recognition of conserved molecules generically known as Microbe-Associated Molecular Patterns (MAMPs). This recognition is mediated by Pattern-Recognition Receptors (PRRs) at the plasma membrane. PRRs can be activated with purified synthetic MAMPs to elicit plant immunity prior to bacterial infection. Such activation of plant immunity elicits changes in the metabolite composition of the LA [12], which in turn, suppress the expression of *Pst* DC3000 T3SS and coronatine biosynthesis genes, thus compromising *Pst* DC3000 virulence and infectivity [12–14]. Altogether, these studies demonstrate that plant defense responses drive changes in the composition of LA metabolites that inhibit *Pst* DC3000 growth and prevent the onset of plant disease. Notwithstanding the relevance of these discoveries, they likely represent only a glimpse of the contribution of LA metabolites to plant immunity.

The complex composition of the LA and the dynamic changes in the concentration of metabolites that take place during the course of infections [15–17] have hindered efforts to define which plant metabolites, and at which concentrations, would have a positive or a negative impact on *Pst* DC3000 infections. Nevertheless, amino acids and sugars present in the LA are emerging as important regulators of *Pst* DC3000 virulence within the LA. This seems especially true for *Pst* DC3000, as bacterial supplementation with glutamine, serine, or valine, decreased bacterial virulence and leaf colonization [12], while aspartate had the opposite effect [13,14]. Like aspartate and other organic acids, glucose was reported to have a positive impact in bacterial virulence gene expression and leaf colonization [14].

Recently published studies have used bacterial gene expression profiling to understand how the LA environment affects *Pst* DC3000 growth and virulence on a global scale [8,9]. These studies showed that MAMP-elicited immunity could suppress the expression of bacterial virulence genes through metabolite deprivation, as previously reported by Anderson and colleagues [13] for aspartate and other organic acids. In addition, Nobori and colleagues [9] found that plant immunity also suppresses the expression of genes encoding bacterial ribosomal proteins, suggesting that MAMP-treated plants may broadly impact *Pst* DC3000 protein synthesis as well. While both studies provided evidence of *Pst* DC3000 virulence suppression, they reached dissimilar conclusions regarding the plant metabolites that may restrict *Pst* DC3000 infections. It may be the case that relying solely on transcriptomics data may not fully capture *Pst* DC3000 metabolic adaptations to the plant environment.

To overcome these potential limitations, we have generated an ensemble of **ge**nome-scale **n**etwork **re**constructions (GENREs) and hypothesize that metabolic modeling could inform of yet undescribed metabolic shifts that multi-omics alone cannot contextualize. GENREs, and their corresponding modeling counterpart called **ge**nome-based **m**odels (GEMs), have emerged as a powerful tool for predicting metabolic phenotypes and gene essentiality [18]. GENREs are originally built from genome annotations and are curated with various forms of evidence, including *in vitro* metabolic demands, protein homology, and literature research [19]. To fill pathway gaps and increase the predictive power of metabolic models, Medlock et al developed AMMEDEUS [20], an algorithm that creates several slightly different models and calculates the likelihood of each model to produce growth. Overall, AMMEDUES lowers the uncertainty in the predictions made by each single model.

Gene expression data can be overlaid on a GENRE blueprint to make integrative predictions of metabolic activity. Multiple algorithms for integrating gene expression data with GEMs have been developed, each of which makes assumptions about the relationship between gene expression and reaction activity [21]. While algorithms like GIMME [22] only assess metabolic flux by overlaying gene expression on to the network, RIPTiDe also considers the overall cost and feasibility of including a reaction based on gene expression [23].

Here we present a metabolic model of *Pst* DC3000, iPst19, using the well-annotated and characterized genome originally assembled by Buell and colleagues [24]. Through literature mining, sequence homology comparisons, and ensemble gap-filling [20], we have iteratively curated the draft reconstruction to be more akin to *Pst* DC3000 biology. Leveraging the unbiased systems-level view, iPst19 made new and insightful predictions that were confirmed experimentally *in vitro* and *in planta*.

## Results

### iPst19, an ensemble of genome-scale metabolic models

**Fig 1:**
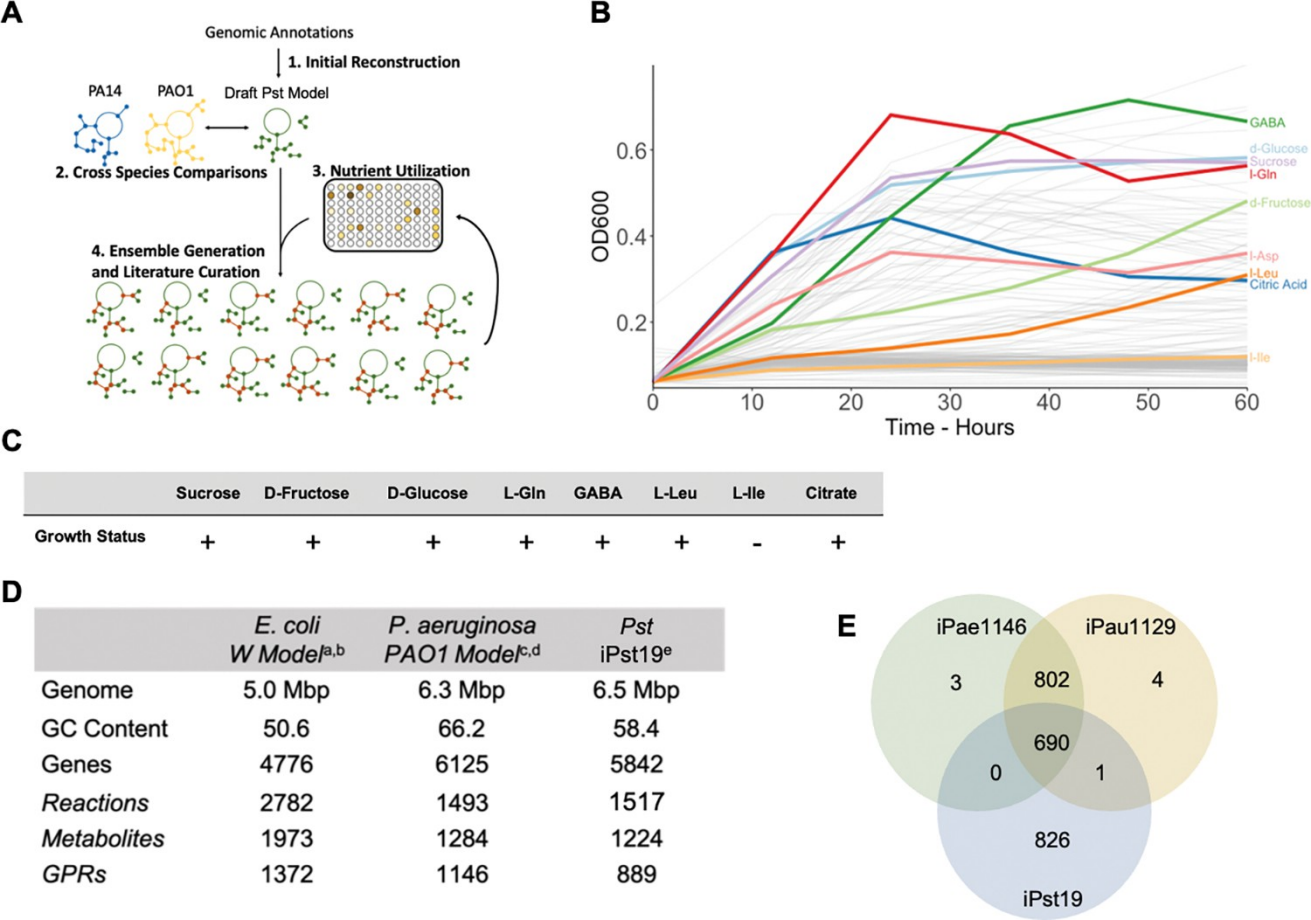
Construction and validation of iPst19. (A) Depiction of the steps followed to construct iPst19, including the initial reconstruction from gene annotations, comparison to other Pseudomonas models, integration of single carbon source growth data, and iterations of gap-filling and manual curation. (B) *Pst* DC3000 grown in 190 single carbon source media over 60 hours with constant agitation. Shown in color are metabolites found in the leaf apoplast of Arabidopsis plants. (C) In silico growth simulations output obtained with iPst19 when the indicated metabolites are used as a single carbon source. (D) Comparison of the size of well-curated models and the corresponding genomes. Genome information for *Escherichia coli* (a) *Pseudomonas aeruginosa* (c) and *Pseudomonas syringae* (c) were obtained from publicly available genome assemblies. The *E. coli* (b) and *P. aeruginosa* models were obtained from the BiGG Models platform at the University of California, San Diego (http://bigg.ucsd.edu). (E) Number of shared and unique reactions within metabolic network reconstructions for *P. aeruginosa* strains PA01 (iPae1146) and PA14 (iPau1129), and *P. syringae* DC3000 (iPst19).

A draft reconstruction of the *Pst* DC3000 GENRE was generated using ModelSEED [25] and genome data from Buell and colleagues [24]. We added additional reactions and corresponding gene-protein-reaction rules (GPRs) to the draft reconstruction using homologous comparison with the published Pseudomonas GEMs iPae1146 and iPau1129 representative of *Pseudomonas aeruginosa* strains PAO1 and PA14, respectively. The draft reconstruction of iPst19 comprised 519 genes to which 355 homologous genes were added based on the interspecies comparison (see Fig 1A step 1: initial reconstruction). The draft was further curated through ensemble gap-filling (Fig 1A). An approach called **A**utomated **M**etabolic **M**odel **E**nsemble-**D**riven **E**limination of **U**ncertainty with **S**tatistical learning (AMMEDEUS) was used to fill the gaps in the original GEM [20]. Briefly, AMMEDEUS incorporates traditional gap-filling of metabolic models based on *in vitro* growth data on single carbon sources (SCS), identifying reactions that collectively carry the minimal amount of flux necessary to produce biomass *in silico* in each condition. AMMEDEUS captures more potential solutions to the objective function (biomass production) by creating an ensemble of metabolic networks that all satisfy the objective function. Because there are little increased capabilities for solving the objective function beyond 100 unique networks [20], we have used 100 networks to form an ensemble of networks called iPst19. To create a carbon utilization list for AMMEDEUS, we performed SCS growth phenotyping using Biolog phenotype microarrays PM1 and PM2a plates (Hayward, CA). We grew *Pst* DC3000 in each of 190 SCS in quadruplicate and recorded the optical density at 600nm (OD600) at 0, 12, 24, 36, 48, and 60 hours (Fig 1B and S1 Dataset). To only include high-confidence growth conditions for gap-filling, we only considered conditions that resulted in a max OD600 greater than 0.3 after subtracting the 0-hour baseline. Most of the metabolites found in the LA that *Pst* DC3000 is known to be unable to use as a SCS (e. g. isoleucine) fell below the 0.3 threshold. As false positives could be more detrimental than false negatives to the overall predictive power of the model, the rather conservative value of 0.3 OD600 ensures that no false positives would be included. Following AMMEDEUS, gap-filled reactions that presented the most uncertainty for producing flux through the biomass function in rich medium were assessed for literature support in *Pst* DC3000 and other Pseudomonads. Reactions with literature evidence were added to the reconstruction. This iterative process was completed three times and rendered 15 genes that were added to the 874 metabolism genes that currently form the 100-member ensemble iPst19 (Fig 1D). Several amino acids and sugars produced significant growth over 60 hours of incubation (Fig 1B). Of special interest were plant metabolites previously shown to have an impact on *Pst* DC3000 infections [4,12,13,26]. GABA, a highly abundant amino acid in the LA of tomato plants, produced robust growth, as did L-glutamine, sucrose, and D-glucose (Fig 1B). We then assessed the *in silico* biomass production on SCS to preliminarily validate the ensemble. Of the highlighted substrates in Fig 1B, all *in silico* simulations were able to predict that they support *Pst* DC3000 growth *in vitro* (Fig 1C). The resulting ensemble of models comprises 1517 unique reactions and 1224 unique metabolites, while each individual member of the ensemble contains 1330 +/- 7 reactions, 1215 +/- 2 metabolites, and 889 GPRs. iPst19 is similar in size to the well-curated *Pseudomonas aeruginosa* GEM for strain PA01 [27], and it is smaller than the gold-standard *E*. *coli* W GEM [28], likely due to the depth of experimental support available for *E*. *coli* (Fig 1D). We produced a draft model with 519 genes obtained from *Pst* DC3000 annotated sequences and added 355 homologous genes based on the interspecies comparison. Of the total 1517 reactions carried out by the 889 GPRs, 826 were unique to iPst19, and 690 were shared with both iPae1146 and iPau1129 model for *P*. *aeruginosa* strains PA01 and PA14 strains, respectively. While iPst19 shared 1 reaction with iPau1129, none was shared with iPae1146 only (Fig 1E and S2 Dataset). The large number of unique reactions present in iPst19 compared to iPae1146 and iPau1129 correlates well with the large number of unique genes in *Pst* DC3000 compared to all members of the Pseudomonas genus. Indeed, genome complexity and variability in protein coding sequences across all Pseudomonas species likely explains the complexity and diversity of metabolic plasticity found in the entire group [29].

### Gene expression-constrained metabolic flux highlights the role of leucine catabolism under growth-restrictive conditions imposed by plant immunity

**Fig 2:**
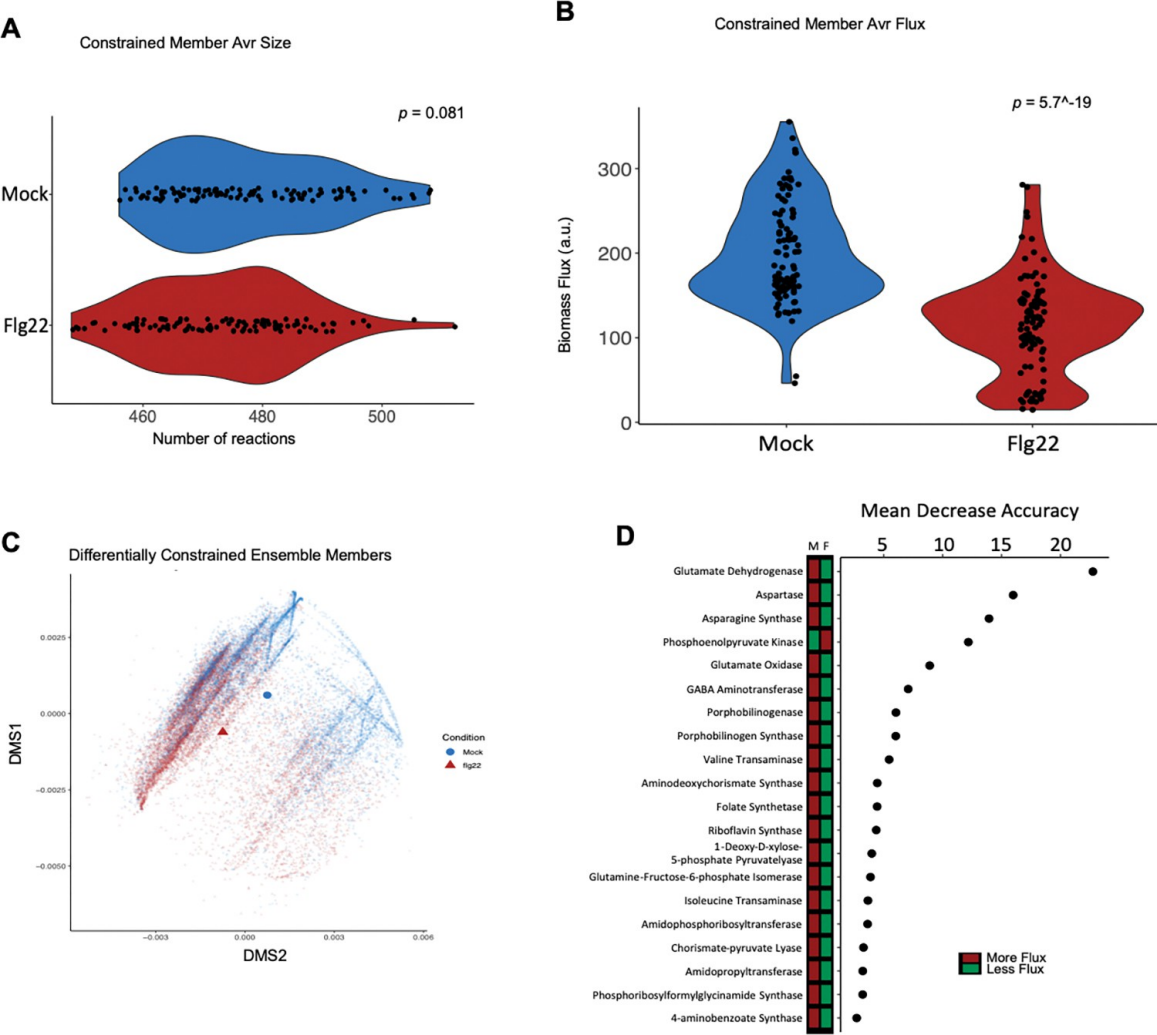
Gene expression-constrained iPst19 highlights the relevance of amino acid metabolism. RNAseq datasets were integrated into iPst19 to constrain fluxes of reactions using the RIPTiDe integration algorithm. In blue or red are shown data obtained from bacteria recovered from mock-treated (Mock) or pre-immunized (PI) plants 5h post inoculation. P-value derived from Wilcoxon test. (A) Reactions within each constrained ensemble member, separated by condition. (B) Average biomass for each constrained member, derived from 1000 flux variability analysis solutions for the biomass reaction per ensemble member. (C) Non-metric multi-dimensional scaling plot of 100 sub-sampled flux variability analysis fluxes from each reaction shared across all conditional and constrained ensemble members. The centroids for each condition are shown as a blue circle and a red triangle. (D) Twenty most influential reactions assessed as mean decrease accuracy (as a percent) of a random forest analysis. The flux difference is highlighted in the bar to the left of the plot. Breiman and Cutler data perturbation method was used as measure of importance.

Integration of transcriptomic profiles into a GEM can contextualize complex relationships between genes and corresponding metabolic pathways [18]. To study the metabolic changes that *Pst* DC3000 experiences in the LA, we use the RIPTiDe transcriptomic integration method [23] to constrain iPst19 metabolic flux. To this end, we used a previously published *in planta* gene expression dataset of *Pst* DC3000 obtained 5h post-inoculation into leaves of Arabidopsis plants that have been mock-treated or pre-immunized with MAMPs 24 hours earlier [9]. Of the 889 genes included in iPst19, 250 were significantly up or downregulated in *Pst* DC3000 between the two contrasting plant environments (S3 Dataset). The contextualized members in each model had the same number of reactions regardless of the condition (Fig 2A), yet the average biomass flux across all 100 members was significantly different between the two conditions tested (Fig 2B). We then used non-metric multidimensional scaling (NMDS) to assess overall differences in flux variability analysis (FVA) solutions. There was a large degree of overlap within the ordination plot at the center top area. However, there were also distinct patterns of reaction flux between ensemble members constrained with each transcriptomics dataset. While a cluster of reactions associated with bacteria recovered from mock-treated plants can be appreciated on the top right quadrant of the plot, the lower left quadrant mostly contains enzymatic reactions associated with bacteria recovered from pre-immunized plants (Fig 2C). With the gene expression-constrained metabolic flux, we identified reactions that carried the most disparate flux between the two contrasting conditions analyzed: mock-treated plants, where *Pst* DC3000 grows aggressively, and pre-immunized plants, where *Pst* DC3000 grows modestly. Random forest analysis revealed that reactions involving glutamate, aspartate, GABA, and BCAA metabolism, ranked among the twenty reactions with the most influential and determinant fluxes shared across all 100 contextualized and condition-specific ensemble members (Fig 2D). Glutamate-related metabolic reactions were particularly abundant in the top twenty hits, with eleven reactions directly or tangentially related to glutamate metabolism. Since the role of glutamate in *Pst* DC3000 colonization of Arabidopsis has already been described [30], the following sections in this study focus on understanding how BCAAs metabolism connects with the pathogenesis of *Pst* DC3000 in *Arabidopsis*.

### iPst19 predicts the essentiality of the *liu* operon to metabolize leucine in growth-restricting conditions

**Fig 3:**
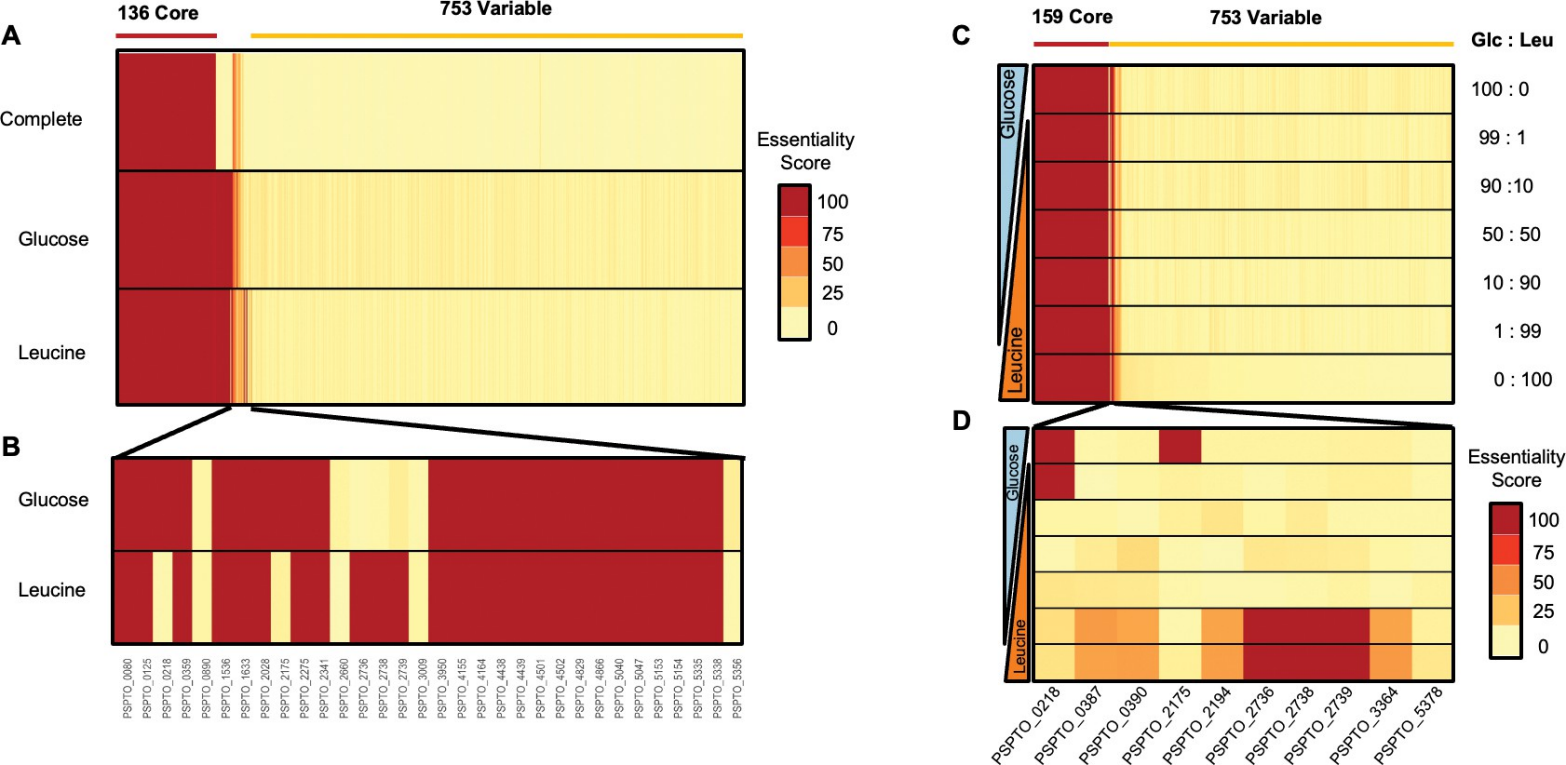
iPst19 predicts genes of the *liu* operon as essential for Leu catabolism. Each of the 889 genes present in iPst19 was removed from a total of 100 ensembles one at a time, and final biomass production was assessed. (A) Predicted essentiality in each medium is given out of 100 ensemble members. An essentiality score of 100 indicates essentiality in all 100 ensemble members, while a score of 0 indicates non-essentiality in all 100 ensembles. iPst19 detected 136 shared predicted essential genes across all tested conditions. (B) Twenty-three predicted glucose- and leucine-specific essential genes are shown, five of which have varying degrees of essentiality depending on the carbon source used: while one ammonium transporter (PSPTO_0218) and one iso-propyl-malate dehydrogenase (PSPTO_2175) were essential to metabolize glucose, three genes of the *liu* operon (PSPTO_2736, PSPTO_2738, and PSPTO_2739) were essential to use leucine as the sole carbon source. (C) Predicted gene essentiality changes with mixed carbon sources where iPst19 gene essentiality was simulated in conditions where carbon sources were restricted to an uptake rate of 10 mM/g dry weight. The carbon composition of the Glc and Leu simulated media is indicated to the right as a percentage. All genes present in iPst19 were simulated for essentiality. (D) The most dissimilar essential genes in D-glucose and L-leucine simulated medium.

In addition to amino acids with known roles in *Pst* DC3000 pathogenesis [12,13,30], the random forest assessment predicted an important role for BCAAs metabolism (Fig 2D), which was never studied in *Pst* DC3000 before. Valine and isoleucine would be a natural choice for testing gene essentiality, yet neither support *Pst* DC3000 growth as a SCS (Fig 1B) [4,12]; thus, leucine was used instead. To understand the biological function of these metabolic signatures, we sought to run an *in silico* genetic screen to identify genes essential to the utilization of glucose and leucine. Glucose and leucine were tested to serve two purposes: while glucose alone produces robust growth experimentally, is implicated in the colonization of mock-treated plants [14], and lacks a nitrogen moiety, leucine is the only BCAA that supports modest growth experimentally and provides a nitrogen moiety that should relieve iPst19 reliance on other sources of nitrogen (e. g. ammonium). We then tested gene essentiality in three different media formulations: a complete medium (where the medium is defined by the transport reactions present within the ensemble), a glucose minimal medium, and a leucine minimal medium. Within an ensemble, gene essentiality varies depending on the substrates available for growth simulation. Due to the gap-filling process used for optimizing iPst19, most of the 100-member ensembles have a unique architecture. Therefore, we simulated single gene knockouts across the 100-member ensemble and scored essentiality as the proportion of members of the ensemble that predict a given gene as essential depending on the carbon source available. From a total of 889 genes, the analysis identified 136 predicted essential genes necessary for biomass production in a rich (complete) medium (Fig 3A). In addition, we identified 28 substrate-specific predicted essential genes (Fig 3B). While twenty-three genes were common to both glucose and leucine SCS media, five were differentially essential. The glucose minimal medium rendered two genes differentially essential from the leucine minimal medium: PSPTO_0218, an ammonium transporter encoding gene, and PSPTO_2175, a gene coding for the iso-propyl-malate dehydrogenase activity that catalyzes one of the first steps in BCAAs biosynthesis. The leucine minimal medium rendered three additional genes predicted to be essential: PSPTO_2736, PSPTO_2738, and PSPTO_2739. The three genes show high sequence homology to the leucine catabolic genes *liuD*, *liuB*, and *liuA*, respectively.

In Pseudomonas, the *liu* operon is conserved across multiple species, including *Pst* DC3000, and shows a high degree of ORFs reshuffling across different bacterial species [31]. Some genes in the operon are induced in the presence of BCAAs [32,33]. Upstream of the enzymatic activities encoded by genes in the *liu* operon, BCAAs degradation requires α-ketoacid dehydrogenase enzymatic complexes composed of three subunits (E1, E2, and E3), each with different reaction specificities. In *Pseudomonas putida*, the leucine catabolism begins with the conversion of leucine into 4-methyl 2-oxopentanoate, a reaction performed by a BCAA-aminotransferase. In *Pst* DC3000, this enzyme is encoded by PSPTO_1332. The next step in leucine degradation requires branched-chain keto acid dehydrogenase (BCKDC) enzymes. While in *P*. *putida* these enzymes are encoded by the *bkd* operon [34], the *Pst* DC3000 genome lacks an operon encoding BCKDCs. BCKDC enzymes, together with those of the 2-oxoglutarate dehydrogenase complex (ODHC) and the pyruvate dehydrogenase complexes (PDC), belong to the α-ketoacid dehydrogenase protein family and share high percentages of identity with one another [35,36]. Notwithstanding the lack of a *bkd* operon, *Pst* DC3000 can use leucine as a SCS (Fig 1B). Hence, we hypothesize that ODHC or PDC enzymes could take 4-methyl 2-oxopentanoate to produce isovaleryl-CoA, which is then further catabolized to acetyl-CoA and acetoacetate by the enzymes encoded by *liuA*, *liuD*, and *liuC* (S1 Fig). To identify enzymes with BCKDC-like activity in *Pst* DC3000, we use the amino acidic sequences of enzymes encoded by the *P*. *putida bkd* operon as a query in BLASTp (https://blast.ncbi.nlm.nih.gov/). The search yielded one ODHC-E1 component encoded by PSPTO_2199, one ODHC-E2 component encoded by either PSPTO_2200 or PSPTO_5006, and one ODHC-E3 component encoded by PSPTO_2201 (S1 Table), suggesting that enzymes of the ODHC complex could be part of the leucine degradation pathway and serve function equivalent to those of the BCKDC complex in *P*. *putida*.

Growth assessment in SCS media provides valuable information on metabolite utilization but does not inform of the sensitivity of the *in silico* screening when more than one SCS is available. To test if the model would respond to nutritional changes in a predictable manner, we simulated growth and generated a gene essentiality profile in conditions where D-glucose and L-leucine were available in varying concentrations. The total availability of mixed substrates in the media was maintained at 10 mM/gDW. In a mixed substrate with 99% glucose and 1% L-leucine, gene essentiality matches predicted gene essentiality in D-glucose alone. Similarly, with 1% D-glucose and 99% L-leucine, the essentiality profile resembles that of L-leucine alone. As expected, the essentiality profile of 50% D-glucose and 50% L-leucine and those with 10% of a second substrate included fewer genes than either single substrate (Fig 3C). The essentiality of genes was not a binary output immediately alleviated by the introduction of the second metabolite. Instead, there is a predicted threshold at which leucine alleviates the need for glucose-only essential genes and *vice versa* (Fig 3D). Since these genes are predicted to be essential only when leucine content reaches 99% of the carbon source in the medium, our results suggest that the concentration of leucine alone would not explain the expression patterns of *liuA* and *liuD in planta* (S3 Dataset). In addition, the data suggest that BCAA degradation may not contribute significant amounts of acetyl-CoA to the tricarboxylic acid (TCA) cycle. Instead of supporting bacterial growth, *liuA* and *liuD* may play a regulatory role during the infection process by modulating the levels of leucine and other BCAA that serve as signals to adjust bacterial growth to changing environmental conditions in the LA.

### BCAAs impact virulence gene expression *in vitro* and *in planta*

**Fig 4:**
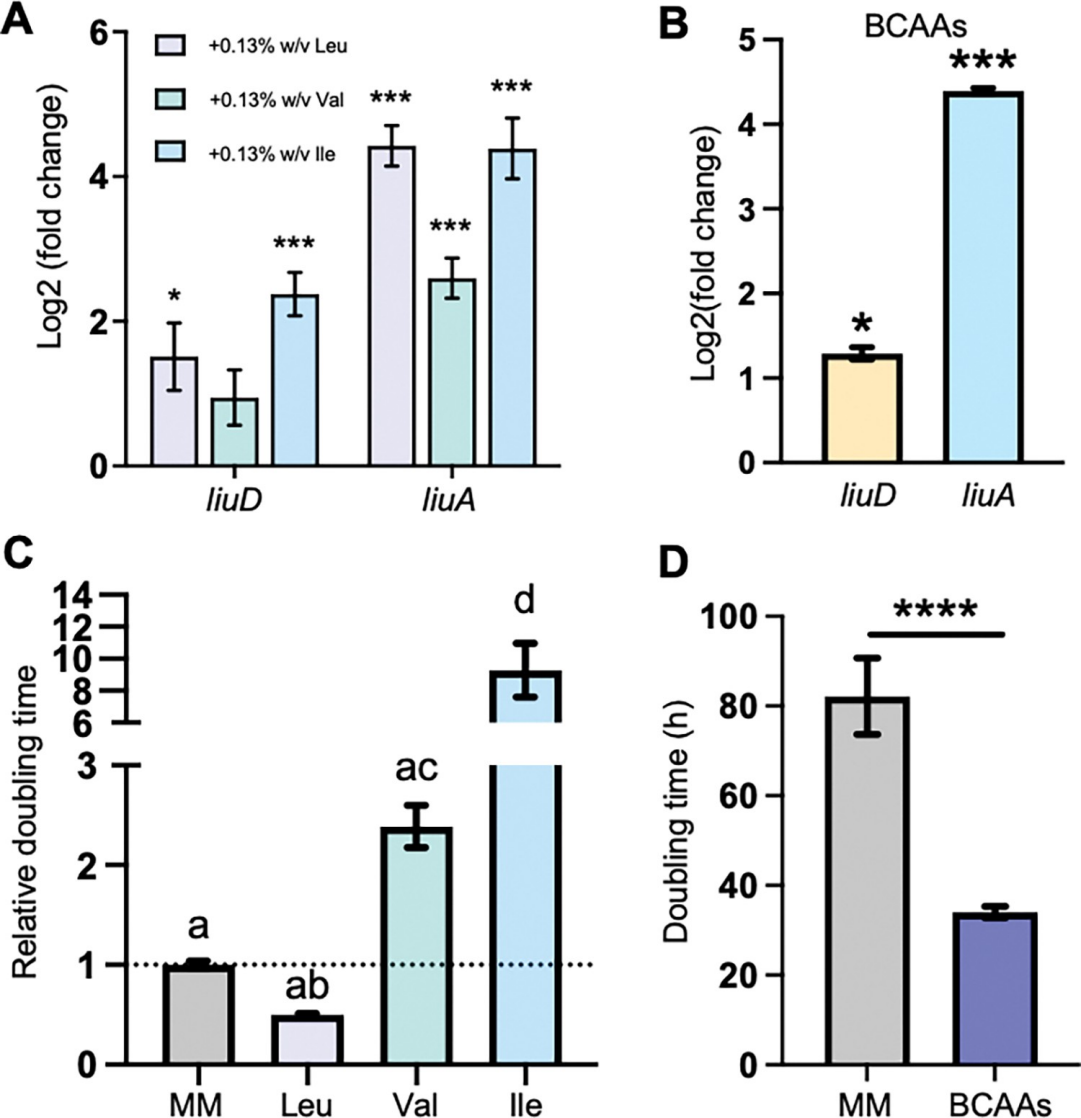
BCAAs levels control leucine catabolism and *Pst* DC3000 growth in vitro. A) Transcript abundance of *liuD* and *liuA* in bacteria exposed to 0.13% w/v Leu, Val, or Ile in hrp-inducing minimal medium (HMM) normalized to non-supplemented minimal medium (N = 5 independent cultures). B) Transcript abundance of *liuD* and *liuA* in bacteria exposed to 0.13% w/v each BCAA in HMM normalized to non-supplemented HMM (N = 5 independent cultures). C) Bacterial growth in MM9 without supplementation or with 0.13% w/v Leu, Val, or Ile (N = 7–15 independent cultures). D) Bacterial growth in minimal media without supplementation or with 0.13% w/v of each BCAA (N = 15 independent cultures). A, B: Student’s *t*-test from relative control, * p < 0.05, *** p < 0.001. C: One-way ANOVA. D: Student’s *t*-test, **** p< 0.0001.

*P*. *aeruginosa* controls leucine levels by a feedback loop where high leucine concentration suppresses leucine biosynthesis and induces leucine degradation, while low leucine concentration has the opposite effect [31,33,37]. The *in planta* expression of *liuA* and *liuD* (S3 Dataset), as well as the iPst19 gene expression-constrained metabolic flux (Fig 2), suggests that leucine, and potentially other BCAAs whose synthesis and degradation are controlled by similar mechanisms [38], accumulate to higher levels in bacterial cells that have been inoculated into pre-immunized plants compared to those inoculated into mock-treated plants. To understand how an excess of BCAA could impact *Pst* DC3000, we supplemented minimal medium with BCAAs, individually or combined, and assessed bacterial growth and gene expression. Supplementation with 0.13% w/v of either leucine, isoleucine, valine, or the three BCAAs combined, induced the expression of both *liuA* and *liuD* compared to non-supplemented bacteria (Fig 4A and 4B). These data support the *in silico* essentiality pattern depicted in Fig 3. While we have not tested the essentiality of the genes directly, both are highly induced in a BCAA-rich medium. *liuA* and *liuD* transcriptional responses were specific to BCAAs, as supplementation with 0.15% w/v Gln and Ser had the opposite effect on their expression (S2 Fig). While leucine had a positive impact on *Pst* DC3000 growth, shortening the doubling time by half, both isoleucine and valine inhibited bacterial growth (Fig 4C). When combined, however, the growth-promoting activity of leucine relieved the growth inhibitory effect of isoleucine and valine (Fig 4D), suggesting that the three BCAAs serve coordinated functions controlling bacterial metabolism. This is consistent with previous studies showing that changing levels of BCAAs play a regulatory role in bacterial growth and virulence in several gram-positive and gram-negative pathogenic bacteria [39].

**Fig 5:**
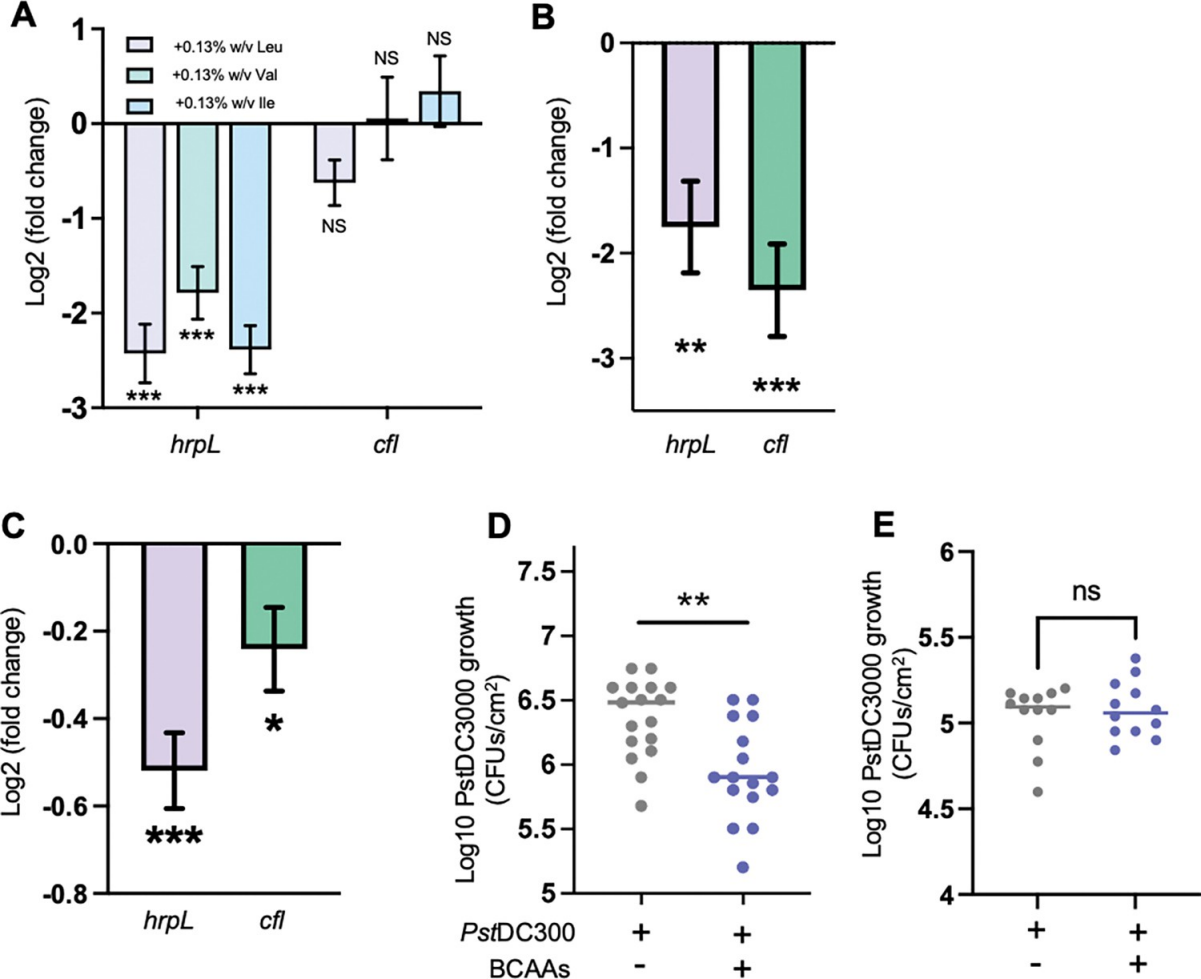
High levels of BCAAs impact the expression of virulence and compromise *Pst* DC3000 colonization of the leaf apoplast. A) Transcript abundance of *hrpL* and *cfl* in bacteria exposed to hrp-inducing minimal medium (HMM) supplemented with 0.13% w/v of Leu, Val, and Ile in minimal media normalized to non-supplemented HMM (N = 5 independent cultures). (B) *hrpL* and *cfl* expression in bacteria exposed to HMM supplemented with 0.13% w/v each BCAA normalized to non-supplemented bacteria (N = 5 independent cultures). C) *in planta hrpL* and *cfl* expression bacteria co-infiltrated with 0.13% w/v each BCAA and normalized to bacteria with 5 mM MES alone. (N = 6 pooled samples of 2 plants each). D) PstDC3000 growth 72 HPI in naïve Arabidopsis plants. Bacteria were suspended in 5 mM MES pH 5.7 or 10 mM each BCAA in 5 mM MES pH 5.7 and infiltrated into leaves (N = 17 individual plants). E) *Pst* DC3000 growth 3 HPI in naïve Arabidopsis plants. Bacteria were suspended in 5 mM MES pH 5.7 or 0.13% w/v each BCAA in 5 mM MES pH 5.7 and infiltrated into leaves (N = 12 individual plants) A, B, C: Student’s *t*-test against control condition, ** p < 0.01, *** p < 0.001. D, E: Student’s *t*-test, ** p< 0.01.

As the onset of bacterial virulence is significantly delayed in pre-immunized plants, we sought to test potential connections between BCAAs metabolism and virulence in *Pst* DC3000. To that end, we assessed the expression of virulence marker genes in BCAAs supplemented minimal medium. While BCAAs induced the expression of *liuA* and *liuD*, both of which were previously shown to be more represented in *Pst* DC3000 isolated from pre-immunized plants (Fig 4A and S3 Dataset), the same amino acids suppressed the expression of the T3SS master regulator gene *hrpL* and the coronatine biosynthesis gene *cfl* when supplemented individually (Fig 5A) or combined (Fig 5B) *in vitro*. A similarly low *hrpL* and *cfl* expression was observed at 3 h post inoculation (HPI) when *Pst* DC3000 was co-infiltrated with BCAAs in naïve plants (Fig 5C). In line with mounting evidence suggesting that virulence expression plays an essential role in the early stages of infection [8,9,12], naive plants that are otherwise susceptible to *Pst* DC3000 infections were able to significantly suppress bacterial growth 72 HPI when co-infiltrated with BCAAs (Fig 5D). Importantly, the number of viable bacteria detected 3 HPI was similar in BCAA-supplemented and non-supplemented bacteria (Fig 5E), suggesting that BCAAs do not have an immediate toxic effect that would explain the lower bacterial infection titer 72 HPI (Fig 5D). Worth noting, the expression of the salicylic acid and the jasmonic acid plant defense marker genes *Pathogenesis-Related gene-1 (PR1)* and *Vegetative Storage Protein-2* (*VSP2*) [40,41], respectively, was not induced by the infiltration of BCAAs, suggesting that the reduced growth of BCAA-supplemented *Pst* DC3000 is likely due to the direct suppressing effect of BCAAs on bacterial virulence expression and not to an induced plant immunity elicited by BCAAs (S3 Fig). Altogether, these data suggest that intracellular levels of BCAAs limit the ability of *Pst* DC3000 to infect Arabidopsis leaves without causing bacterial toxicity or inducing plant immunity.

## Discussion

### BCAAs impact *Pst* DC3000 virulence

BCAAs serve a central role in integrating extracellular and intracellular cues to maximize bacterial growth and survival [39,42]. BCAAs have been shown to modulate the growth and the virulence of bacterial pathogens, including *E*. *coli* and *Salmonella enterica*, through the leucine-responsive regulatory protein (Lrp) transcriptional activity [39]. In *E. coli*, leucine binding to Lrp changes Lrp conformation and its affinity for DNA binding sequences, thus modulating the expression of different sets of genes [43,44]. Recent studies have provided evidence that BCAAs and Lrp also play an important role in the pathogenesis of plant bacterial pathogens [45,46]. In *Xanthomonas citri* pv. *citri*, leucine degradation takes place via the acyl-CoA carboxylase complex (ACC) encoded by the *acc* operon. Open reading frames in the *X*. *citri acc* operon show an organization similar to that of the *liu* operon in *P*. *aeruginosa*. In addition, *AccC* and *AccD* show 53% and 70% identity with *liuD* (PSPTO_2736) and *liuB* (PSPTO_2738), respectively, strongly suggesting that both the *X*. *citri acc* operon and the *Pst* DC3000 *liu* operon are functionally equivalent and that both contribute to leucine catabolism. Mutant strains for *accC* and *accD* showed attenuated growth on citrus plants, suggesting that leucine catabolism in *X*. *citri* is important for virulence expression and pathogenicity *in planta* [45]. The modulation of virulence gene expression exerted by BCAAs on Lrp is both ways, positive on certain genes and negative on others [47]. In the plant pathogen *Erwinia amylovora*, Lrp is necessary to express virulence genes that control motility and synthesis of exopolysaccharides, a function that becomes apparent when BCAA levels are low [46]. Similarly, in the corn pathogen *Pantoea stewartia*, Lrp is necessary to induce disease symptoms in the plant, indicative of successful virulence expression [48]. The evidence contributed by Tomassetti et al. [45], Schachterle and Sundin [46], and Bartholomew et al. [48] suggest that leucine degradation, and more broadly, BCAAs degradation, plays a positive role in virulence induction by lowering intracellular levels of BCAAs and allowing Lrp to transcribe virulence genes. The molecular mechanisms by which BCAAs impact gene expression in *Pst* DC3000 remain unknown. It would be expected, however, that BCAAs bind Lrp and directly modulate its activity on gene expression as previously described for other bacterial species [42]. The data presented in this study (Fig 5) show that the supplementation of *Pst* DC3000 with BCAAs was sufficient to suppress the expression of *hrpL* (Figs 4A and 5A) suggesting that high BCAAs levels could interfere with the Lrp-mediated transcription of virulence genes. Conversely, these data suggest that low concentrations of BCAAs could allow Lrp to express virulence genes. Since high BCAAs levels suppress *Pst* DC3000 virulence gene expression (Fig 5) and induce *liuA* and *liuD*, lowering intracellular BCAAs levels could contribute to re-directing Lrp activity toward the expression of virulence genes to counteract the overall virulence-suppressing environment imposed by inducible plant immunity. The levels of BCAA co-infiltrated with *Pst* DC3000 into the leaf apoplast (Fig 4) are higher than the endogenous levels found in Arabidopsis [12]. However, as shown previously for other amino acids [12], it would be expected that most of the supplemented BCAAs will quickly be taken up by plant cells, lowering the effective concentration to which bacteria are exposed. In addition, as BCAAs need to be transported into *Pst* DC3000 to impact gene expression likely via modulating Lrp activity, a high extracellular concentration is necessary to produce an increase in effective intracellular levels. Whether the increased concentration of BCAA detected in the leaf apoplast of pre-immunized plants [12] translates into an increased in BCAAs intracellular concentration and changes in Lrp transcriptional activity is unknown.

In *E*. *coli* and several other bacterial species, BCAAs biosynthesis is controlled by negative feedback inhibition, with valine and isoleucine having a major effect, and leucine a minor to no effect [49–51]. The data presented in this study (Fig 4) show that both isoleucine and valine inhibit bacterial growth *in vitro*, suggesting that feedback inhibition could holt BCAAs biosynthesis, protein synthesis, and eventually cell division. Leucine not only did not inhibit bacterial growth but also relived isoleucine and valine mediated inhibition, suggesting that an excess of isoleucine and valine may primarily inhibit the synthesis of leucine, which is rescued by adding leucine to the milieu. Cell division arrest is often associated with increased levels of (p)ppGpp, which in turn induces virulence gene expression in *Pst* DC3000 [52]. Thus, if valine and isoleucine were able to arrest protein synthesis, we would have expected a positive impact on the expression of virulence genes, which was not the case (Fig 5). In addition, since the combination of the three BCAAs shortened the doubling time in liquid medium (Fig 4D) it would be unlikely that the three BCAAs have a direct growth suppressing effect in leaves (Fig 5D). Whether these responses to BCAAs are mediated by the transcriptional regulator Lrp is still unknown. Further studies will be necessary to understand how intracellular levels of BCAAs impact the expression of virulence genes.

### BCAAs catabolism relieves plant immunity-mediated suppression of virulence gene expression

In *P*. *putida*, the *bkd* operon contributes a set of reactions that produce substrates for the enzymes encoded by the *liu* operon (S1 Fig). Notwithstanding that *Pst* DC3000 lacks a *bkd* operon and possesses a re-arranged *liu* operon, *Pst* DC3000 is still able to grow when leucine is supplied as a SCS (Figs 1B and 4). These data suggest that other enzymes compensate for the loss of the branched-chain ketoacid-dehydrogenases (BCKDH) encoded by the *bdk* operon. In *P*. *aeruginosa* and *P*. *putida*, the pyruvate dehydrogenase complex (PDC) and the oxoglutarate dehydrogenase complex (ODHC), share significant homology with the BCKDH enzymes [35,36,53]. In *Pst* DC3000, PSPTO_3860, PSPTO_5005, PSPTO_5006, and PSPTO_2201 encode the PDC enzymes, while PSPTO_2199, PSPTO_2200, and PSPTO_2201 encode the ODHC enzymes. Importantly, enzymes in these two complexes show a degree of promiscuity in substrate utilization, suggesting that they could also contribute to BCAAs catabolism in the absence of a canonical *bkd* operon [54]. Indeed, ODHC enzymes encoded by *Pst* DC3000 showed the highest identity to those encoded by the *bkd* operon in *P*. *putida* (S1 Fig and S1 Table). It is likely through these enzymes that *Pst* DC3000 still metabolizes leucine as a SCS (Fig 1B).

Importantly, *Pst* DC3000 likely synthesizes BCAAs via enzymes encoded by the *ilv* and *leu* operons [24]. Notwithstanding the role of BCAAs on virulence gene expression, *Pst* DC3000 still responds to changes in the extracellular levels in BCAAs (Fig 4) and hence is susceptible to environmental perturbation. In a previous study, we have shown that pre-immunized plants delay the onset of *Pst* DC3000 virulence via the accumulation of virulence-suppressing amino acids, especially glutamine, serine, and valine [12], suggesting that the induced expression of *liuA* and *liuD* (S3 Dataset) would be part of a mechanism that counteracts the suppression of virulence gene expression.

Like *Pst* DC3000, *P*. *aeruginosa* uses BCAA levels to adjust the expression of virulence genes [55]. Under low iron conditions that mimic the infection of animal hosts, *P*. *aeruginosa* induces BCAAs catabolism and virulence [55]. This enhanced catabolism leads to a drop in intracellular concentrations of BCAAs that, interestingly, is not compensated by enhanced uptake of BCAAs from the environment [55], suggesting that *P*. *aeruginosa* relies more on BCAAs synthesis than on uptake to better control virulence expression and growth. Iron deprivation was a major signature that emerged from the global gene expression analysis of *Pst* DC3000 inoculated in pre-immunized plants [9]. Several iron-responsive genes were included in iPst19 (S3 Dataset), yet through the gene expression-constrained flux analysis, reactions associated with iron metabolism did not rank in the top twenty most determinant reactions shared across all 100 constrained members of iPst19 (Fig 2 and S3 Dataset).

Several factors, including iron deficiency and the increased availability of certain amino acids in the LA of pre-immunized plants [12] could induce BCAAs degradation to overcome the plant immunity-mediated suppression of virulence. Further studies will be necessary to elucidate how the plant environment influences intracellular levels of BCAAs in *Pst* DC3000.

### The power of computational models to uncover the molecular underpinnings of plant-bacteria interactions

The integration of transcriptomic data to constrain iPst19 metabolic flux revealed no significant differences in the size of condition-constrained models, yet clear differences in optimal FVA-derived biomass solutions with mock-constrained members producing larger biomass fluxes (Fig 2A and 2B). NMDS of all shared reaction fluxes revealed a shared metabolic state between the conditions with outlying differences. In particular, amino acid metabolism seemed to play a disproportionate role in differentiating between conditions (Fig 2C and 2D). Among 1294 *Pst* DC3000 genes differentially expressed in mock-treated or pre-immunized plants [9], 250 were metabolic genes present in iPst19 (S3 Dataset). Yet, the constrained metabolic flux analysis indicated that the associated reactions contributed by most of these 250 metabolic genes were not contrasting enough to warrant further study (Fig 2D). Importantly, iPst19 rendered a hierarchy of contrasting metabolic reactions that do not correlate with the magnitude of gene expression changes (Fig 2D) and allows us to draw further conclusions from existing data. In other words, iPst19 predictions are, to some extent, influenced by gene expression, yet gene expression is not the only contributing factor when assessing differences between conditions. Rather, the overall architecture of each of the 100 members, and thus the architecture of the iPst19 ensemble, plays a major role in determining the importance of metabolic differences.

Importantly, we have also presented a new method for assessing gene essentiality. By leveraging the differential architecture across the 100 members of the iPst19 ensemble generated by AMMEDUES, we can now generate an essentiality probability score that could, more accurately, predict gene essentiality (Fig 3). Furthermore, the gene expression integration and the gene essentiality screen predicted the relevance of specific bacterial genes and pathways that were overlooked in previous studies. Owed to the relatively conservative OD600 of 0.3 used as a cutoff to build a list of growth-producing metabolites (Fig 1B), and the approach used for gap-filling the models with AMMEADUES, which eliminates complexity to generate robust networks, it seems plausible that bacterial metabolic genes that play important but more subtle roles in infections may have been overlooked in this study. While gene expression analysis reveals important facets of the *Pst* DC3000-*Arabidopsis* interactions, our findings demonstrate the power of using metabolic computational models to contextualize transcriptomic data. Integration of *in planta Pst* DC3000 transcriptomics has highlighted the role of the BCAAs on virulence gene expression. These predictions were tested and confirmed experimentally, lending support to the strong predictive power of iPst19. In depth genetic and molecular studies will be needed to address the role of the *liu* operon and *lrp* in the regulation of virulence gene expression. In addition, other transcriptomics datasets could be similarly contextualized using iPst19 to further understand how *Pst* DC3000 metabolism contributes to infecting host plants.

## Materials and methods

### *Pst* DC3000 genome annotations and cross-species comparison

The *Pst* DC3000 genome assembly used was generated by Buell and colleagues [24]. The draft GENRE was generated using ModelSEED v2.1 [25] and the RAST database [56], and further optimized using the COBRApy toolbox [57]. The *Pst* DC3000 GENRE was refined using cross-species homologous comparisons with two *Pseudomonas aeruginosa* models, iPau1129 and iPae1146. Protein alignments between *Pst* DC3000 features and *P*. *aeruginosa* features included in their respective GENREs were made with DIAMOND [58]. Comparisons that yielded a significant (e-value < 0.0001) were queried for associated reactions in the *P*. *aeruginosa* GENREs and subsequently added to the *Pst* DC3000 GENRE with the significantly matching homolog that had functional literature support.

### Ensemble generation

A full description of the ensemble process and justification was previously published by Medlock and Papin [20]. iPst19 was generated from the cross-species compared draft reconstruction with integration from single carbon source utilization data (see Biolog growth assays). Each substrate that supported *Pst* DC3000 growth, as defined by significantly different maximum measured OD_600nm_ from the negative control, was compiled into a randomly ordered list for use in gap-filling the draft reconstruction using AMMEDEUS. The specific order of metabolites is essential during the gap-filling process, as only the most parsimonious use of the metabolite will result in the addition of a given reaction to the model. If a metabolite can be utilized with the metabolic infrastructure already in place, no new reactions will be added; otherwise, the reactions that add the minimum amount of flux will be added to the model to make use of the metabolite. Only when the draft reconstruction is gap filled and can satisfy the fixed-growth constraint of the biomass function and minimize the fluxes through all other reactions on all *in vitro* growth-producing metabolites (as empirically assessed with Biolog plates), is it then considered a member of the ensemble. The process repeats, starting with the draft reconstruction and a shuffled order of the growth-producing metabolites. As a result of this reshuffling, every member has a slightly different architecture and may produce different biomass fluxes on simulated media. Manual curation was conducted during each round of ensemble gap filling for the most uncertain reactions introduced by AMMEDEUS, where there was disagreement among members regarding whether the reaction should be included. Extensive literature research was utilized to inform the curation process. This resulted in the addition of 16 reactions and 15 genes of previously high uncertainty with curated literature support for a particular architecture within the members. The full repository is available at 10.5281/zenodo.7942779.

### Biomass quantification

*Pst* DC3000 was grown as described below in “bacterial growth conditions”. Liquid cultures were centrifuged at 3500 RPM for 10 minutes. Growth media was removed, the bacterial pellet was washed twice using sterile water. The pellet was resuspended in 10 mL sterile water and immediately frozen and lyophilized. Samples were split for each quantification assay, ensuring the samples would be matched for the different quantification protocols. Dry weights of each fraction were recorded. Total protein was quantified using a standard Bradford’s assay on lysed cells [59]. DNA and RNA were extracted from lyophilized cells using protocols for Gram negative bacteria [60, 61]. Both RNA and DNA were quantified using spectrophotometry. All quantifications were normalized to the total dry weight of bacterial cells. Other coefficients were determined through literature refinement and equations of closely related Pseudomonads. Coefficients for the biomass equation were determined as a percent of 1 unit of biomass.

### Ensemble single gene deletions

For each of the 100 members in iPst19, every gene within each member was simulated as a loss of function. For reactions with only one gene association, the flux of the reaction became zero. In reactions in which the gene deletion was part of an “and” association, the reaction flux also became zero. The reaction flux was unaltered for reactions where the deleted gene was in an “or” association. Final readouts of objective function flux were assessed: if the reaction flux was zero or near zero (flux<10e-5), the gene was predicted to be an essential gene.

### Ensemble multi-carbon growth media gene essentiality

Multi-carbon media simulations were made from L-leucine and D-glucose combinations as a percent of the total carbon atoms present in the media. Combinations included 100% glucose, 99% glucose:1% leucine, 90% glucose:10% leucine, 50% glucose:50% leucine, 10% glucose:90% leucine, 1% glucose:99% leucine, and 100% leucine.

### Ensemble transcriptomic integration

RNAseq global gene expression profiles of *Pst* DC3000 5h post-inoculation in mock or pre-immunized (MAMP-treated) plants generated by Nobori and colleagues [9] were integrated into iPst19 members using RIPTiDe to generate FVA flux samples [23]. Random forest machine learning was applied to the resultant FVA samples in a ratio of 70% training and 30% learning to query which reactions, shared across all contextualized ensemble members, were most influential for determining bacterial metabolic differences in each environment (mock-treated plants versus and pre-immunized plants). Data Breiman and Cutler data perturbation method was used as measure of importance in random forest analysis [62].

### Bacterial growth conditions

All bacterial strains were grown in liquid King’s B culture at shaking at 230 RPM and 28°C. Bacteria for growth rate assessment and plant infection experiments were taken from fresh LB agar plates (<1 week old) and grown overnight in the liquid media, followed by sub-culturing 10% until mid-exponential phase.

### Plant growth conditions

*Arabidopsis thaliana* Columbia-0 (Col-0) plants were grown in peat pellets with a 12-hour photoperiod at 23°C and 70% humidity. Plants were watered three times a week with Hoagland’s solution for four weeks and then with water for two more weeks. Six weeks old plants were used for infections and gene expression analysis.

### Bacterial gene expression

For *in vitro* bacterial gene expression, overnight cultures were sub-cultured at 10% in fresh KB media, followed by agitation for two hours at 28°C. Bacteria were pelleted via centrifugation and washed three times in sterile water, after which the bacteria were resuspended to the appropriate OD600 and exposed to experimental conditions.

The same method was used to prepare bacteria for *in planta* gene expression assays. The bacteria were resuspended to a final OD600 of 0.2 and pressure-infiltrated into the leaves with a needleless 1 mL syringe. After three hours, infected leaves were harvested and immediately flash frozen in liquid nitrogen; standard RNA isolation from plant tissue or bacteria followed using phenol/chloroform extraction.

### Gene expression analysis

Transcripts of genes were quantified via RT-qPCR as described previously [12]. RNA was extracted from leaves or bacterial liquid cultures via phenol/chloroform extraction and treated with DNase 1 (Promega) to remove gDNA contamination. cDNA was synthesized from normalized RNA input using m-MLV reverse transcriptase (Promega). qPCR was performed in duplicate with at least three biological replicates and normalized using the ΔΔCt method with *recA* as housekeeping gene.

### Primers

A complete list of primers used in this study are available in S2 Table.

### Biolog growth assays

Biolog (Biolog Inc., Hayward, CA) PM1 (Cat #12111) and PM2 (Cat #12112) plates were inoculated with 100 μL of IF0 per well, in which *Pst* DC3000 was suspended at 0.07 OD600. Plates were shaken at 7000 rpm and 28°C for 60 hours. OD600 measurements were taken every 12 hours. Breath-Easy sealing membrane was secured to the plate to ensure gas exchange and prevent evaporation.

### *In planta* bacterial growth assays

*Pst* DC3000 was grown as previously described in bacterial growth conditions. Cells were pelleted in microcentrifuge tubes spun at 10,000 RPM. Pellets were washed and resuspended in sterile water to a final inoculation titer of 0.0002 OD600. Six-week-old *Arabidopsis thaliana* Colombia-0 plants were pressure-infiltrated with a needleless 1 mL syringe. Infections proceeded for 72 hours, after which 8 leaf discs were taken with a hole puncher from 4 infected leaves. Discs were homogenized in 400 μL sterile water, with 2 metal beads in 2 mL round bottom tubes in a QIAGEN Tissue Lyser. Ten-fold serial dilutions were plated on LB agar OmniTray plates. CFUs were counted after 16 hours of incubation at 28°C under a dissecting microscope.

## Supporting information

S1 Fig *Pst* DC3000 tentative leucine catabolic pathway. (TIFF)

S2 Fig *liuA* and *liuD* expression in response to high levels of glutamine and serine. (TIFF)

S3 Fig Gene expression of plant defense markers genes *PR1* and *VSP2*. (TIFF)

S1 Table Tentative *Pst* DC3000 genes encoding BCKDC-like enzymes. (TIFF)

S2 Table DNA oligonucleotides used in this study. (TIFF)

S1 Dataset Shows PstDC3000 bacterial growth in Biolog phenotype microarray PM1 and PM2a plates. These data is presented as a graph in Fig 1B.
(XLSX)

S2 Dataset Shows the metabolic reactions unique to iPst19 and shared with the *Pseudomonas aeruginosa* metabolic models Pae1114 and Pau1129. These data was used to create the Venn diagram shown in Fig 1E.
(XLSX)

S3 Dataset Shows the list and expression values of *Pst*DC3000 metabolic genes used for constraining iPst19 metabolic flux. These gene expression data was integrated into iPst19 using the RIPTiDe transcriptomic integration method.
(XLSX)
