# Supplementary figures and images for: A computational model of Pseudomonas syringae metabolism unveils a role for branched-chain amino acids in Arabidopsis leaf colonization
Source: PLoS Comput Biol. 2023 Dec 27;19(12):e1011651. doi: 10.1371/journal.pcbi.1011651 (PMC10775980; doi:10.1371/journal.pcbi.1011651)

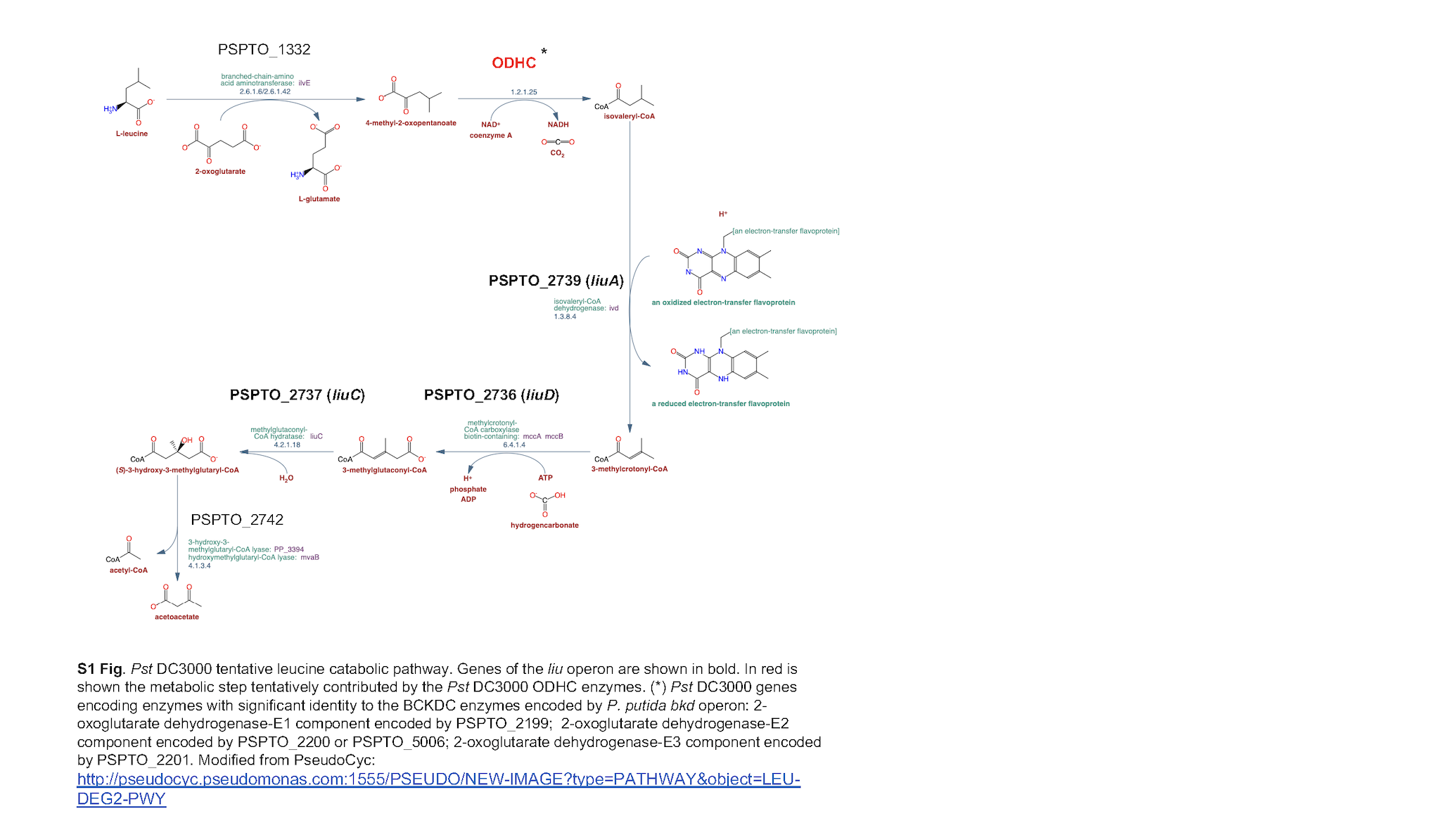

Supplement: S1 Fig — (TIFF) [file pcbi.1011651.s001.tiff]

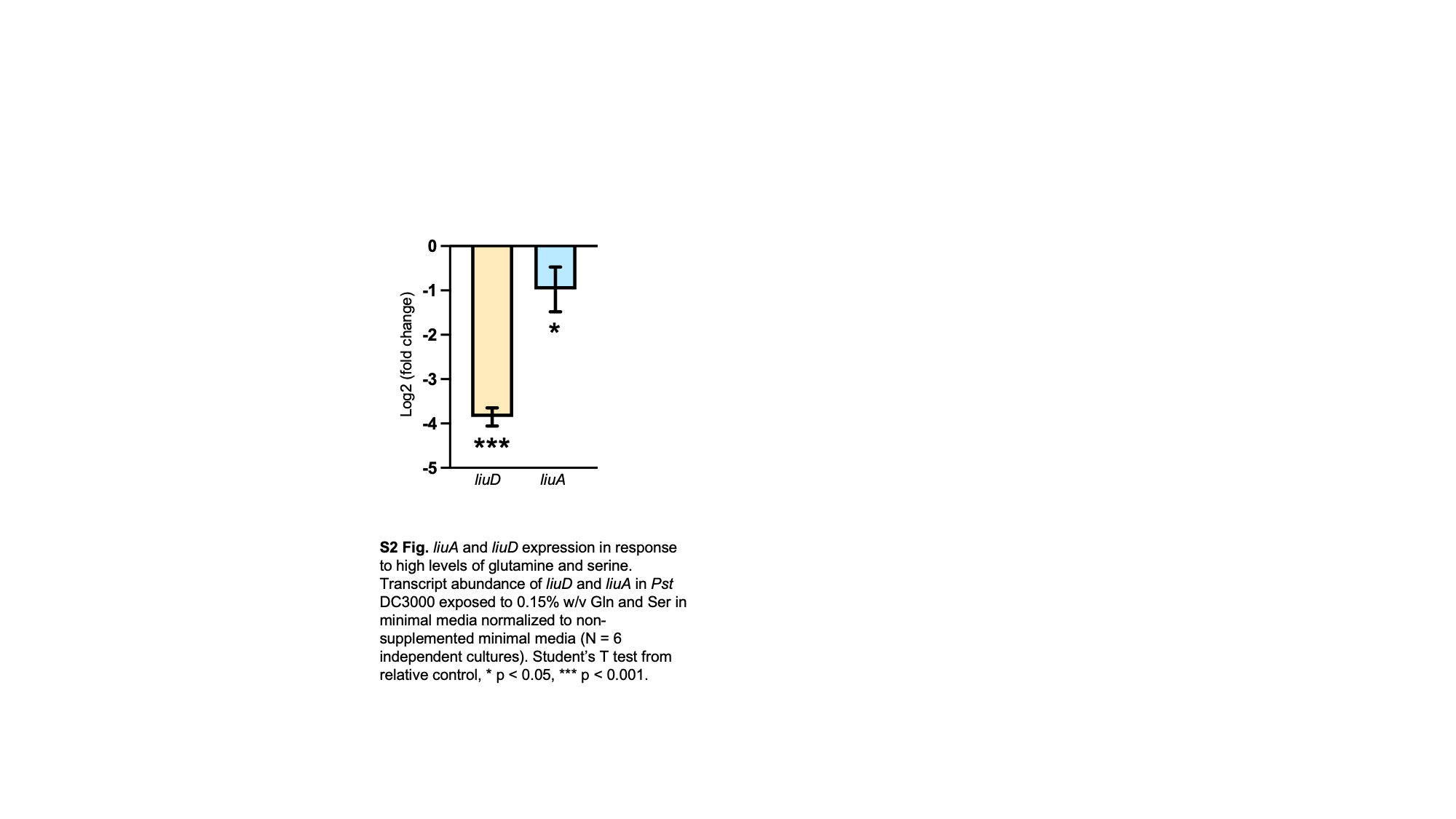

Supplement: S2 Fig — (TIFF) [file pcbi.1011651.s002.tiff]

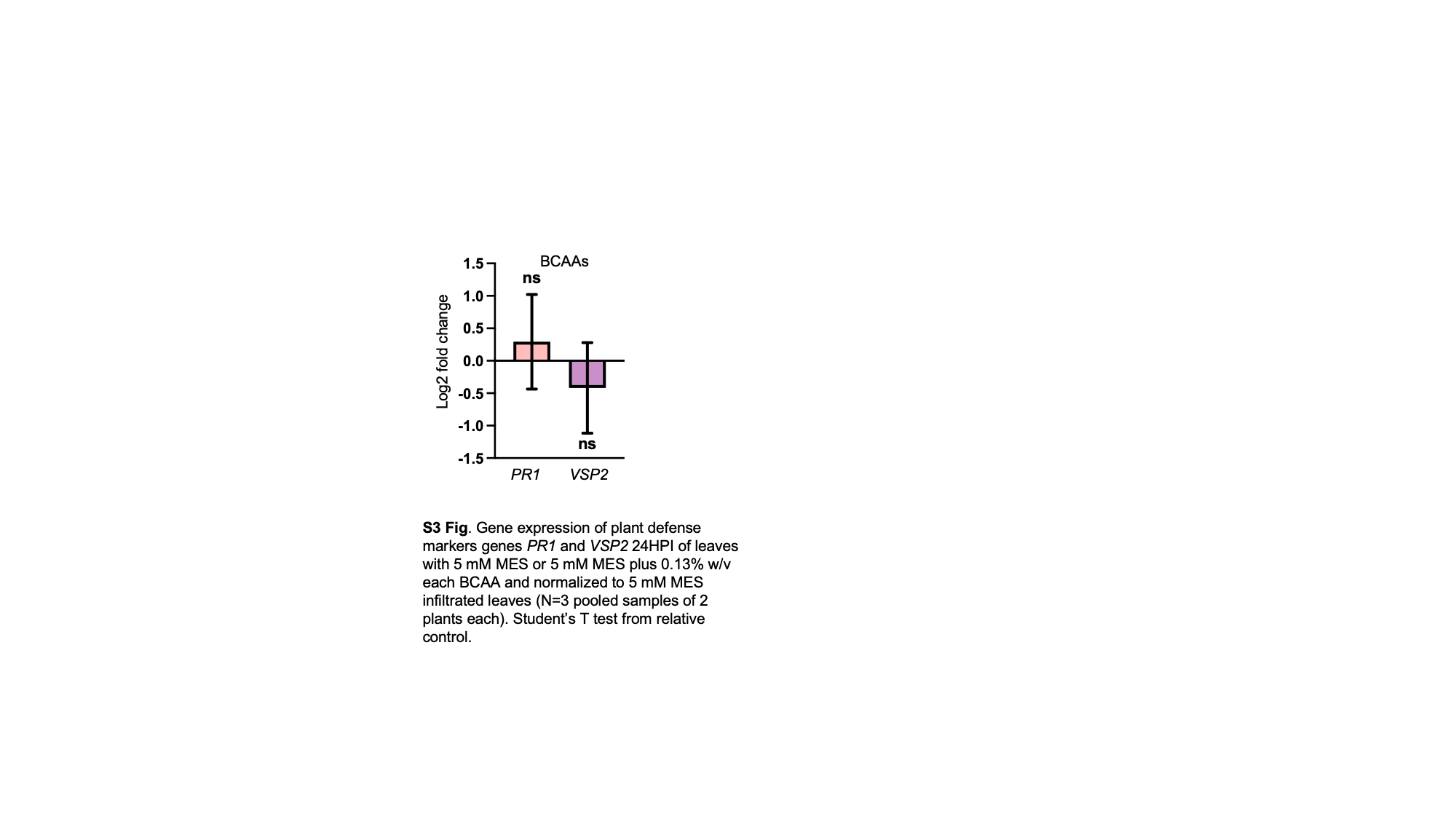

Supplement: S3 Fig — (TIFF) [file pcbi.1011651.s003.tiff]

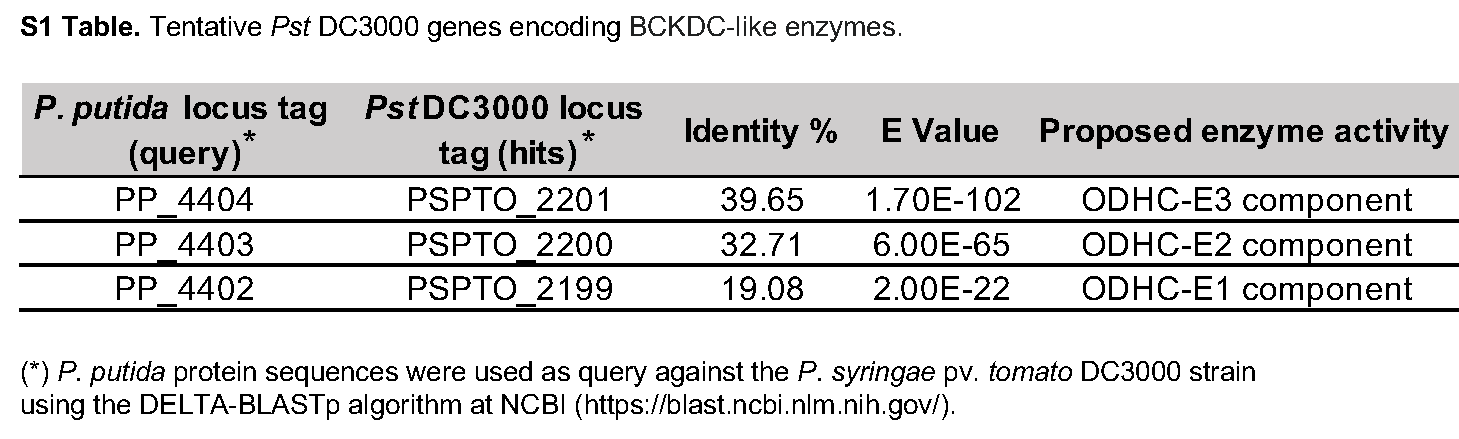

Supplement: S1 Table — (TIFF) [file pcbi.1011651.s004.tiff]

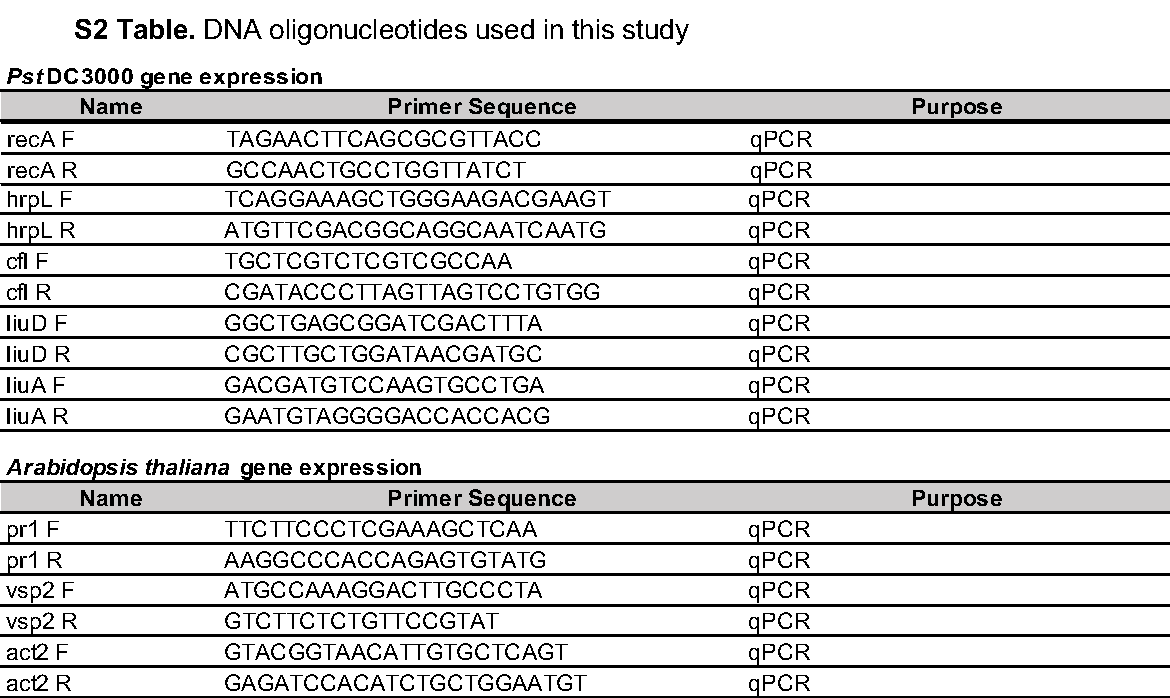

Supplement: S2 Table — (TIFF) [file pcbi.1011651.s005.tiff]
